# In Situ DRIFTS Studies of NH_3_-SCR Mechanism over V_2_O_5_-CeO_2_/TiO_2_-ZrO_2_ Catalysts for Selective Catalytic Reduction of NO*_x_*

**DOI:** 10.3390/ma11081307

**Published:** 2018-07-28

**Authors:** Yaping Zhang, Xiupeng Yue, Tianjiao Huang, Kai Shen, Bin Lu

**Affiliations:** Key Laboratory of Energy Thermal Conversion and Control of Ministry of Education, School of Energy and Environment, Southeast University, Nanjing 210096, China; 101011153@seu.edu.cn (Y.Z.); yuexiupengbg@163.com (X.Y.); 220150460@seu.edu.cn (T.H.); BinLu909@163.com (B.L.)

**Keywords:** in situ DRIFTS, V_2_O_5_-CeO_2_/TiO_2_-ZrO_2_, catalysts, NH_3_-SCR mechanism, NO*_x_*, adsorption

## Abstract

TiO_2_-ZrO_2_ (Ti-Zr) carrier was prepared by a co-precipitation method and 1 wt. % V_2_O_5_ and 0.2 CeO_2_ (the Mole ratio of Ce to Ti-Zr) was impregnated to obtain the V_2_O_5_-CeO_2_/TiO_2_-ZrO_2_ catalyst for the selective catalytic reduction of NO*_x_* by NH_3_. The transient activity tests and the in situ DRIFTS (diffuse reflectance infrared Fourier transform spectroscopy) analyses were employed to explore the NH_3_-SCR (selective catalytic reduction) mechanism systematically, and by designing various conditions of single or mixing feeding gas and pre-treatment ways, a possible pathway of NO*_x_* reduction was proposed. It was found that NH_3_ exhibited a competitive advantage over NO in its adsorption on the catalyst surface, and could form an active intermediate substance of -NH_2_. More acid sites and intermediate reaction species (-NH_2_), at lower temperatures, significantly promoted the SCR activity of the V_2_O_5_-0.2CeO_2_/TiO_2_-ZrO_2_ catalyst. The presence of O_2_ could promote the conversion of NO to NO_2_, while NO_2_ was easier to reduce. The co-existence of NH_3_ and O_2_ resulted in the NH_3_ adsorption strength being lower, as compared to tests without O_2_, since O_2_ could occupy a part of the active site. Due to CeO_2’_s excellent oxygen storage-release capacity, NH_3_ adsorption was weakened, in comparison to the 1 wt. % V_2_O_5_-0.2CeO_2_/TiO_2_-ZrO_2_ catalyst. If NO*_x_* were to be pre-adsorbed in the catalyst, the formation of nitrate and nitro species would be difficult to desorb, which would greatly hinder the SCR reaction. All the findings concluded that NH_3_-SCR worked mainly through the Eley-Rideal (E-R) mechanism.

## 1. Introduction

Generally, nitrogen oxides (NO*_x_*), which may cause environmental problems, such as: Photochemical smog; acid rain; ozone depletion; and, health hazards, are mainly emitted from the industrial combustion of fossil fuels. Therefore, the reduction of NO*_x_* has become an important research field for atmospheric environmental control. Currently, the selective catalytic reduction (SCR) is the most promising method to reduce the emissions of NO*_x_* [1,2]. The temperature window of the traditional V-W (Mo)/Ti catalyst is 300–400 °C, but in some coal-fired power plants, the temperature of exhaust gas is lower. In order to enhance the NO*_x_* conversion rate, the exhaust gases were reheated, which caused a large waste of energy. The narrow temperature window restrained its application. Thus, many researchers redirected their study to focus on the catalyst, which has superior low-temperature activity.

The VO*_x_*/TiO_2_ system for SCR has been studied extensively in the past and a number of reaction mechanisms have been proposed. It is generally accepted that the Brønsted and Lewis acid sites are essential for the reaction mechanism. Topsoe et al. [3] proposed a “Brønsted NH_4_^+^” mechanism over a V_2_O_5_-based catalyst, which has gained the majority of support in the literature. Arnarson et al. [4] observed the SCR reaction over the VO_3_H/TiO_2_ catalyst and demonstrated that the Brønsted acid site served to capture the NH_3_ and increased the NH_4_^+^ stability (increased Brønsted acid strength), which impacted the catalytic rate in a negative direction. Marberger et al. [5] had a similar conclusion for the V_2_O_5_-WO_3_/TiO_2_ catalyst. That is, the Brønsted acid sites hardly contributed to the SCR activity and mainly served as an NH_3_ pool to replenish the Lewis sites. NO reacted predominantly with NH_3_ adsorbed in the Lewis acid sites at low temperatures. SCR reactions over Ce-based catalysts mainly followed two mechanisms, one is the Eley–Rideal mechanism (i.e., the reaction of gaseous NO with adsorbed NH_3_ species), and the other is the Langmuir–Hinshelwood mechanism (i.e., the reaction of adsorbed NO*_x_* with adsorbed NH_3_ species on adjacent sites) [6]. While these two reaction pathways probably do not exclude each other, it is essential to understand whether either or both species are relevant. Vuong et al. [7] reported that NH_3_-SCR proceeded from a Langmuir–Hinshelwood mechanism on bare supports (TiO_2_), while an Eley–Rideal mechanism operated on V-containing catalysts.

In recent years, cerium oxides have attracted extensive attention due to their outstanding oxygen storage-release capacity and excellent redox properties in the low-temperature NH_3_-SCR reactions [8,9,10]. In V/Ce_1−*x*_Ti*_x_*O_2_ catalysts, Ce-O sites are effectively covered by VO*_x_* species, which hinder the formation of surface nitrates and cause the switch in the reaction mechanism. Zhang et al. [11] observed the adsorption and reaction processes in DRIFTS (diffuse reflectance infrared Fourier transform spectroscopy) spectra and concluded that the cis-N_2_O_2_^2−^ formed on CeO_2_ reacted more favorably with NH_3_ than with other nitrate species. Galvez et al. [12] demonstrated that the SCR reaction over activated carbon supported the V_2_O_5_ catalysts (V_2_O_5_/AC) that took place between the adsorbed species of NH_3_ on the Brønsted acid sites, and the NO molecules in the gaseous phase, following an Eley–Rideal (E–R) mechanism. In Yu et al. [13], the study proposed that the SCR reaction over Zr_3_ (PO_4_)_2_/CeO_2_-ZrO_2_ proceeded via the combination of the adjacent, surface N*_x_*O_*y*_ species, and the ads-NH_3_ species by Langmuir-Hinshelwood (L-H) mechanism. Ma et al. [14] also observed the enhanced NH_3_ activation and NO_3_-formation. The latter promoted the reaction of ads-NH_3_ and ads-NO_3_-species for the SCR reaction over N_b_O*_x_*/CeO_2_-ZrO_2_ catalysts—according to the “L-H” mechanism. Getting to know the reaction pathway and proposing reaction mechanisms is helpful in guiding the design and preparation of the catalysts [15].

In our previous study [9,16], a series of 1 wt. % V_2_O_5_-CeO_2_/TiO_2_-ZrO_2_ catalysts with different contents of CeO_2_were prepared by an impregnation method. It was found that the sample of Ce/Ti = 0.2 (the molar ratio) exhibited a favorable performance with a 92% NO*_x_* conversion rate at 250 °C. In addition, the effect of Ce modification on microscopic properties and the catalytic performance of V_2_O_5_/TiO_2_-ZrO_2_ were investigated in more detail. It concluded that the promotional effect of adding Cemainly laid in the intensified interaction between the metal oxide components and the larger amount of Brønsted and Lewis acid sites, as well as the formation of active intermediates (-NH_2_). In this study, we further investigated the NH_3_-SCR mechanism over the optimal 1 wt. % V_2_O_5_-0.2CeO_2_/TiO_2_-ZrO_2_ catalyst, and by carrying out transient activity tests and in situ DRIFTS analyses under various conditions of single or mixing feeding gas and pre-treatment ways, proposed a possible reaction pathway.

## 2. Results and Discussion

### 2.1. Adsorption and Desorption Properties of NO_x_ and NH_3_ on the Catalysts

**Figure 1:**
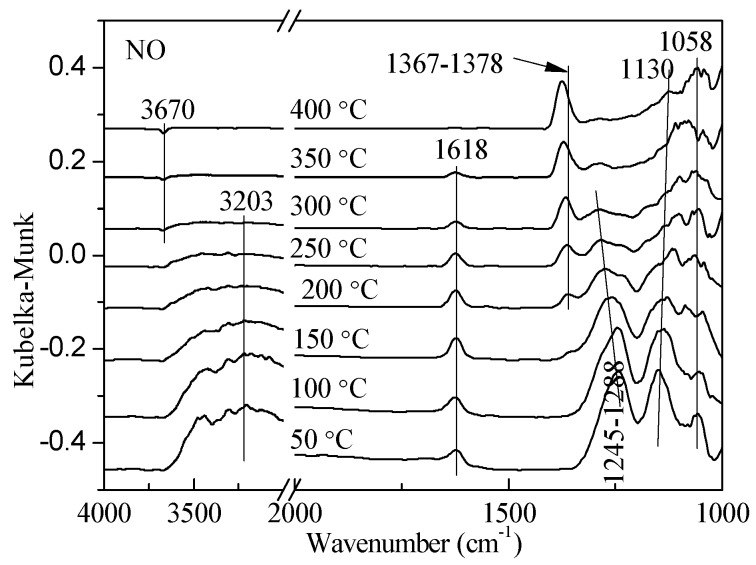
In situ DRIFTS (diffuse reflectance infrared Fourier transform spectroscopy) spectra of NO desorption on 1 wt. % V_2_O_5_-0.2CeO_2_/TiO_2_-ZrO_2_ as a function of temperature after the catalyst was exposed to a flow of 800 ppm NO for 60 min at 25 °C.

The adsorption-desorption behavior of the catalyst is considered to be a crucial step to a heterogeneous catalysis system. To study the desorption status of the reactant gas on the catalyst surface, the desorption of NO on 1 wt. % V_2_O_5_-0.2CeO_2_/TiO_2_-ZrO_2_ was studied. As shown in Figure 1, the band at 3670 cm^−1^ was attributed to O-H, and it decreased with the increased temperature until the negative peak appeared. The band at 3203 cm^−1^ was the result of the hydroxyl vibration. The catalysts contained a little bit of water at a normal temperature, and the water evaporated as the temperature rose; adsorption bands then disappeared. The bands (1618 cm^−1^, 1367–1378 cm^−1^, 1245–1288 cm^−1^, 1130 cm^−1^, and 1058 cm^−1^) were ascribed to the adsorbed NO*_x_*, especially the band of 1618 cm^−1^ which was related to weak adsorption of NO and NO_2_ [16,17,18]. In the case of cis-N_2_O_2_^2−^, bands should appear in the 1300–1400 cm^−1^ [19]. When the temperature exceeded 200 °C, N_2_O_2_^2−^ appeared in the region of 1367–1378 cm^−1^ and the intensity of peaks increased as the temperature rose. This confirmed that it could exist stably on the surface of the catalyst. The band at 1245 cm^−1^ was due to bridging nitrate; the adsorption intensity receded as the temperature rose, and the band region moved to 1288 cm^−1^ with the generation of monodentate nitrate [17,20,21]. Subsequently, this peak disappeared as the temperature reached 400 °C. The band at 1130 cm^−1^ was assigned to nitrosyl NO^−^, which could be oxidized to nitrite and nitrate with the existence of oxygen, and it sharply decreased as the temperature increased [22,23]. The band at 1054 cm^−1^ corresponded to nitrate species, which could exist on the surface of the catalyst stably and was hard to desorb even when the temperature was raised.

**Figure 2:**
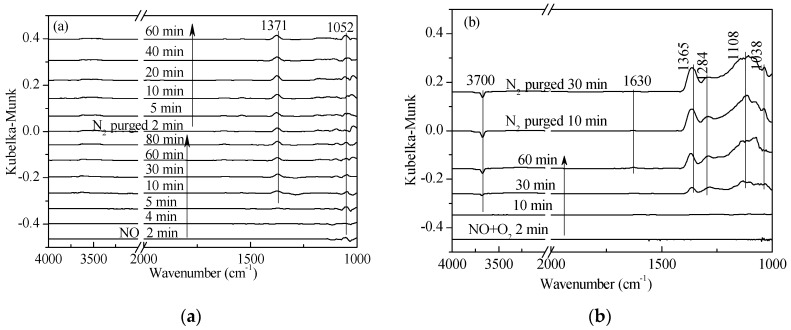
In situ DRIFTS spectra of (**a**) NO adsorption and (**b**) NO + O_2_ adsorption on 1 wt. % V_2_O_5_-0.2CeO_2_/TiO_2_-ZrO_2_catalysts with N_2_ purging for various time at 250 °C after the catalysts were exposed to a flow of 800 ppm NO or 800 ppm NO + 5% O_2_ for 60 min.

As shown in Figure 2a, as N_2_ was steadily purged on, it was clear that the adsorption of NO was very weak at 250 °C, and N_2_O_2_^2−^ and nitrate species only appeared at the band of 1371 cm^−1^ and 1052 cm^−1^. In addition, there was no significant change in peak intensity by increasing the adsorption and desorption time, indicating that it could exist stably on the surface of the catalyst. As shown in Figure 2b, it was observed that the presence of O_2_ obviously strengthened the adsorption intensity of NO*_x_* on the surface of catalysts. After being exposed to NO + O_2_ for 60 min, weak adsorption of NO and NO_2_ appeared at the band of 1630 cm^−1^. The bands at 1365 cm^−1^ and 1108 cm^−1^ could be assigned to cis- and trans-N_2_O_2_^2−^ [19,24], respectively. Simultaneously, the bands at 1284 cm^−1^ and 1038 cm^−1^ were attributed to monodentate nitrate and nitrate species, respectively.

**Figure 3:**
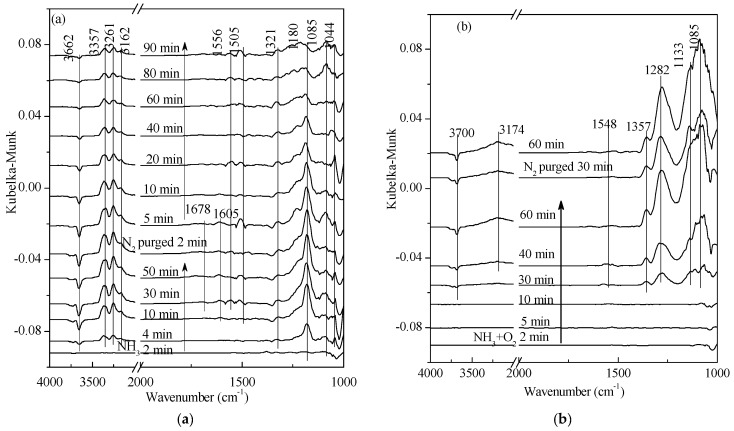
In situ DRIFTS spectra of (**a**) NH_3_ adsorption and (**b**) NH_3_ + O_2_ adsorption on V_2_O_5_-0.2CeO_2_/TiO_2_-ZrO_2_ catalysts with N_2_ purging for various time at 250 °C after the catalysts were exposed to a flow of 800 ppm NH_3_ or 800 ppm NH_3_ + 5% O_2_ for 60 min.

As shown in Figure 3, the adsorption peaks of free O-H appeared at 3662 cm^−1^ and 3700 cm^−1^, and the band at 3100–3400 cm^−1^ was associated with the N-H stretching vibrations, which are linked to Lewis acid sites. The peaks at 1556 cm^−1^, 1548 cm^−1^, and 1505 cm^−1^ corresponded to the formation of intermediate species (-NH_2_) in SCR reactions [21]. In addition, there was no significant change in peak intensity as adsorption and desorption time increased. According to our previous study [16], intermediate species (-NH_2_) were detected above 300 °C over the V_2_O_5_/TiO_2_-ZrO_2_ catalyst. The V_2_O_5_-0.2CeO_2_/TiO_2_-ZrO_2_ catalyst exhibited more -NH_2_ at lower temperatures, which explained its higher activity in comparison to other catalysts. Vuong et al. [11] collected different DRIFTS spectra of bare supports (CeO_2_, TiO_2_ and CeO_2_-TiO_2_) and supported vanadium catalysts (V/CeO_2_, V/CeO_2_-TiO_2_ and V/TiO_2_) at 200 °C. They reported additional bands at 1510–1520 cm^−1^ of NH_2_, which were only observed on pure CeO_2_ and CeO_2_-TiO_2_. It could be speculated that the addition of Ce was the key factor to affect the surface adsorbed NH_3_ species. At the same time, the peaks at 1605 cm^−1^, 1357 cm^−1^, 1321 cm^−1^, 1282 cm^−1^, 1180 cm^−1^, and 1133 cm^−1^ were associated with NH_3_ cooperating vibration—linked to Lewis acid sites [25,26]. According to our previous study [16], with the addition of Ce, the acid sites of the catalysts increased and the optimal V_2_O_5_-0.2CeO_2_/TiO_2_-ZrO_2_ sample possessed the largest amount of surface acid sites, which greatly promoted the SCR reaction. The same trend was observed in Vuong et al. [7]. They demonstrated that the relative amount of Lewis acid sites in the V-containing catalysts decreased in the order V/Ce_0.5_Ti0_0.5_O_2_ > V/CeO_2_ > V/TiO_2_. The band at 1180 cm^−1^ in Figure 3a split into two NH_3_ adsorption peaks (1085 cm^−1^ and 1044 cm^−1^), and the band at 1133 cm^−1^ in Figure 3b corresponded to the peak at 1085 cm^−1^. The band at 1678 cm^−1^ was associated with NH^4+^ symmetric vibration and is linked to Brønsted acid sites [27,28]. Comparing Figure 3a with Figure 3b, it can be found that the presence of O_2_ hindered the adsorption of NH_3_. However, in Figure 3b, after the feeding of NH_3_ + O_2_ was stopped, the intensity of the NH_3_ adsorption peak, linked to Lewis acid sites, was stronger than in Figure 3a. It might have been caused by the re-adsorption of desorbed ammonia or weak ammonia adsorption on Lewis acid sites, because CeO_2_ had the capacity of oxygen storage-release and O_2_ occupied some active sites.

### 2.2. Transient Response Experiment Analysis

**Figure 4:**
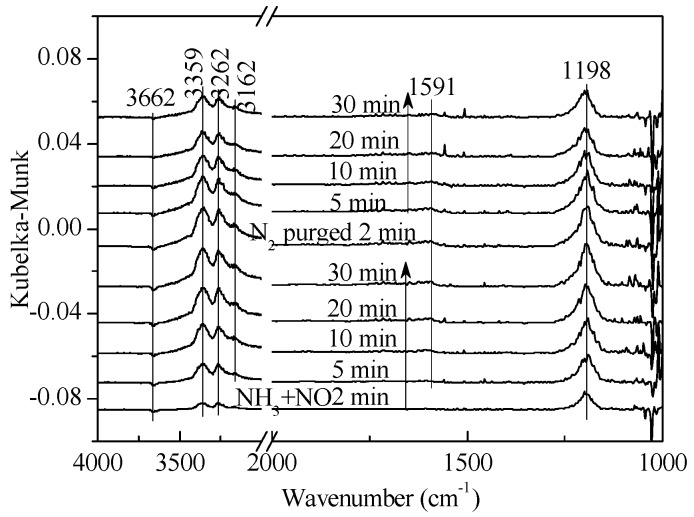
In situ DRIFTS spectra of NH_3_ + NO adsorption on 1 wt. % V_2_O_5_-0.2CeO_2_/TiO_2_-ZrO_2_ catalysts with N_2_ purging for various times at 250 °C after the catalysts were exposed to a flow of 800 ppm NH_3_ and 800 ppm NO for 60 min.

In order to illuminate the difference between NO*_x_* species and explain the SCR reaction mechanism, transient reaction studies by in situ DRIFTS spectra were performed. As shown in Figure 4, the NH_3_ adsorption peak could be found after NH_3_ and NO were introduced for two min. After adsorption was saturated, the bands at 3400 cm^−1^, 3100 cm^−1^, and 1198 cm^−1^ were associated with NH_3_ adsorption and was linked to Lewis acid sites. The intermediate species (-NH_2_) appeared at 1591 cm^−1^, which implied more active intermediates for the NH_3_ oxidation reaction. No obvious NO*_x_* adsorption was observed; it was a preliminary inference that SCR reactions mainly followed from the Eley–Rideal mechanism.

**Figure 5:**
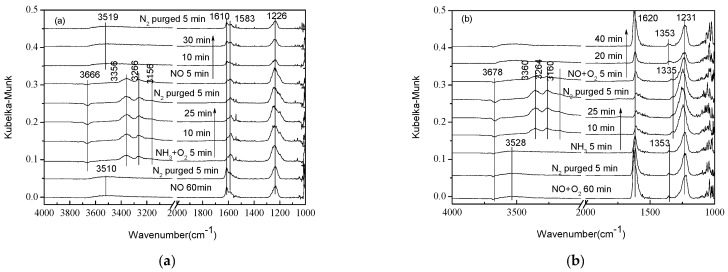
In situ DRIFTS spectra of the transient reactions at 250 °C between (**a**) NO and pre-adsorbed NH_3_ + O_2_ and (**b**) NH_3_, and pre-adsorbed NO + O_2_ species over 1 wt. % V_2_O_5_-0.2CeO_2_/TiO_2_-ZrO_2_ catalysts recorded as a function of time.

As shown in Figure 5, catalysts were exposed to the flow of NO and NO + O_2_ at 250 °C for 60 min. The O-H adsorption peaks appeared at 3510 cm^−1^ and 3528 cm^−1^. The N-H stretching vibration peaks appeared in the range of 3400–3100 cm^−1^ after NO was introduced for two min. However, in Figure 5b, NH_3_ adsorption peaks appeared after NH_3_ was introduced for 10 min in the same region. NO_2_ asymmetric vibration adsorption peaks appeared at 1610 cm^−1^ and 1620 cm^−1^ in Figure 5a,b, respectively. The bands at 1583 cm^−1^, 1226 cm^−1^, and 1231 cm^−1^ were ascribed to bridging nitrates. cis-N_2_O_2_^2−^ appeared at 1353 cm^−1^ in Figure 5b, and it shifted to the region of 1335 cm^−1^ with the introduction of NH_3_, which then weakened the adsorption. When introducing NO + O_2_ again, the adsorption peak recovered to 1353 cm^−1^.

NH_3_ had no obvious influence on the NO*_x_* adsorption peak at 2000–1000 cm^−1^, especially after being exposed to NO + O_2_ where the influence became tinier. After the pre-adsorption of NO, the intensity of the NO*_x_* adsorption peak was obvious, but NH_3_ adsorption could barely be found. With the introduction of NH_3_, the N-H stretching vibration was present in the range of 3400–3100 cm^−1^, as seen in both Figure 5a,b. The results showed that when NO + O_2_ was injected separately, NO + O_2_ occupies SCR active reaction sites and restrains the adsorption of NH_3_, before hindering the SCR reaction. When NO and NO + O_2_ was reintroduced, respectively, peaks located at 3519 cm^−1^ and assigned to O-H adsorption were observed. The intensity of the NO*_x_* adsorption peak had no decrement; on the contrary, NH_3_ adsorption, which was linked to Lewis acid sites, disappeared. These results indicated that the gas-phase NO*_x_* had reacted with NH_3_ on Lewis acid sites, which verified the Eley–Rideal mechanism on catalysts. However, in Chen et al. [29], a Langmuir–Hinshelwood mechanism operated on the CeTi catalyst, and adsorbed NH_3_ and NH_4_^+^ that reacted with NO/O_2_ from the gas phase. Vuong et al. [7] demonstrated that the switch in reaction mechanisms has its roots in the structural differences of catalysts and supports. In V/Ce_1−*x*_Ti*_x_*O_2_ catalysts, Ce-O sites are effectively covered by VO*_x_* species, which hinders the formation of surface nitrates and causes the switch in the reaction mechanism.

**Figure 6:**
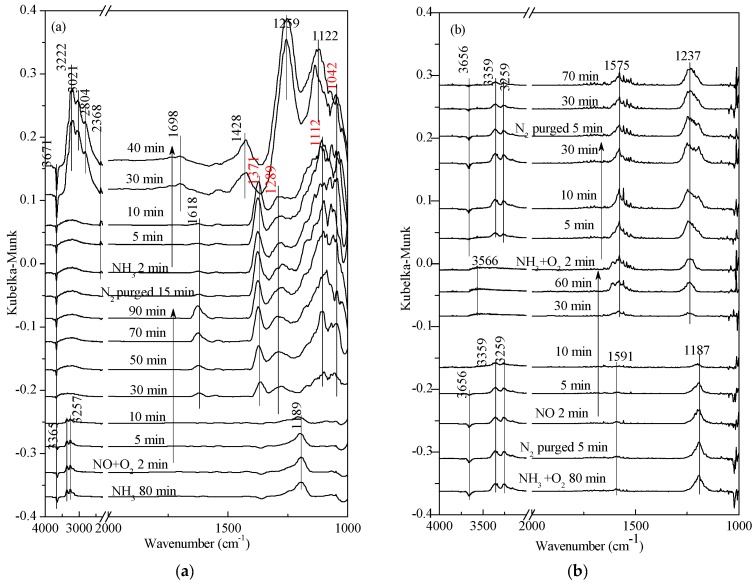
In situ DRIFTS spectra of the transient reactions at 250 °C between (**a**) NO + O_2_ and pre-adsorbed NH_3_, and (**b**) NO and pre-adsorbed NH_3_ + O_2_ species over 1 wt. % V_2_O_5_-0.2CeO_2_/TiO_2_-ZrO_2_ catalysts recorded as a function of time.

As shown in Figure 6a, when NO + O_2_ is introduced, the adsorption peaks at the region of 3400–3100 cm^−1^ and 1189 cm^−1^ disappeared, while the O-H adsorption peak (1618 cm^−1^), the N_2_O_2_^2−^ adsorption peak (1371 cm^−1^ and 1112 cm^−1^), and the nitrate species peak (1024 cm^−1^) appeared. When NH_3_ was introduced again, the NO_2_ adsorption peak disappeared. Moreover, a strong adsorption of NH_3_ appeared at the region of 3400−3100 cm^−1^ and 1259 cm^−1^. Brønsted acid adsorption appeared at 1698 cm^−1^ and 1428 cm^−1^ and considerably intensified, while other NO*_x_* adsorption had no obvious change.

As shown in Figure 6b, NO was introduced after being exposed to NH_3_ + O_2_. Bridging nitrate and monodentate nitrate appeared at 1575 cm^−1^ and O-H vibration appeared at 3566 cm^−1^. When NH_3_ + O_2_were introduced again, the O-H vibration became stronger and the NH_3_ adsorption peak at the region of 3400–3100 cm^−1^ was heavily weakened, as compared with that in Figure 6a. As a result, it can be concluded that O_2_ reacted with NO first.

**Figure 7:**
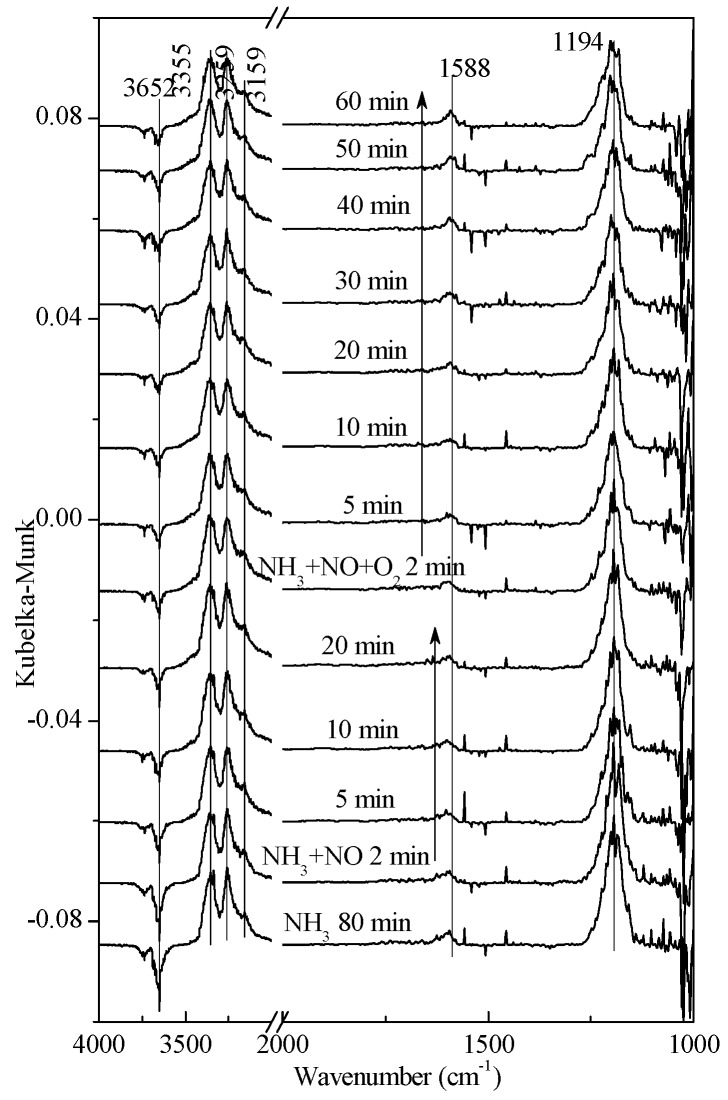
In situ DRIFTS spectra of NH_3_ + NO + O_2_ pre-adsorption transient reaction at 250 °C over 1 wt. %V_2_O_5_-0.2CeO_2_/TiO_2_-ZrO_2_ catalysts recorded as a function of time.

In Figure 7, NH_3_ was introduced first, and then NO was introduced in combination with NH_3._ Lastly, O_2_ was also introduced with NH_3_, NO, and O_2_ being presented at the same time. In these three different atmospheres, the intensity of NH_3_ adsorption on Lewis acid sites had no change. Meanwhile, the active intermediate species of -NH_2_ appeared at 1588 cm^−1^, indicating that NH_3_ molecules continued to be adsorbed on the catalytic surface with the process of reaction. The stable existence of intermediate species (-NH_2_) explained the high SCR activity of the V_2_O_5_-0.2CeO_2_/TiO_2_-ZrO_2_ catalyst at low temperatures. Simultaneously, the intensity of the O-H negative peak receded gradually, which might have been caused by the H_2_O produced in the SCR reaction.

### 2.3. Steady-State Response Experiments

**Figure 8:**
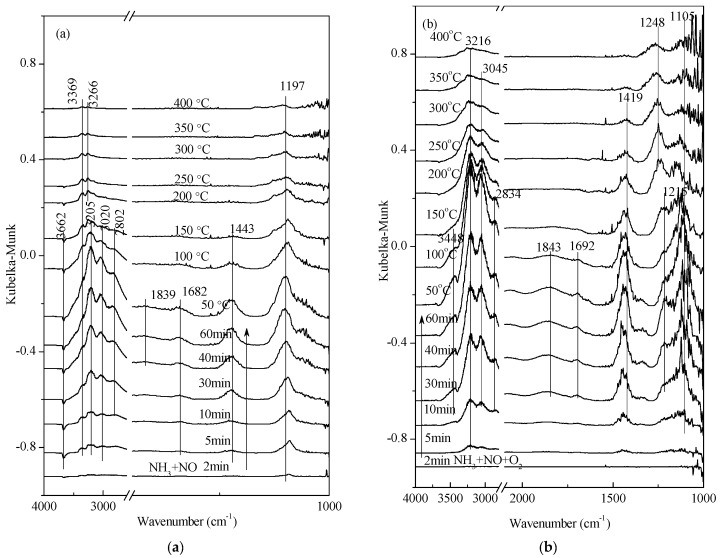
In situ DRIFTS spectra of (**a**) NH_3_ + NO desorption and (**b**) NH_3_ + NO + O_2_ desorption on 1 wt. % V_2_O_5_-0.2CeO_2_/TiO_2_-ZrO_2_ as a function of temperature after the catalyst was exposed to a flow of 800 ppm NO, 800 ppm NH_3_, and 5% O_2_ for 60 min at 25 °C.

As shown in Figure 8, catalysts were saturated at 25 °C after 60 min pre-adsorption. -NO_2_ adsorption appeared at 1839 cm^−1^ and 1843 cm^−1^. The bands at 1692 cm^−1^, 1682 cm^−1^, 1443 cm^−1^, and 1419 cm^−1^ were associated with NH_4_^+^ adsorption, linked to Brønsted acid sites, and the bands of 3400–3100 cm^−1^,1197 cm^−1^, and 1215 cm^−1^ were associated with NH_3_ adsorption, linked to Lewis acid sites. As shown in Figure 8b, N_2_O_2_^2−^ species appeared at 1106 cm^−1^ with the presence of O_2_, which indicated that the existence of O_2_ would promote NO adsorption. Comparing Figure 8a with Figure 8b, NH_3_ adsorption became much stronger with the existence of O_2_. At the same time, the combination of NO*_x_* and NH_3_ appeared at 1248 cm^−1^, and N_2_O_2_^2−^ species decreased with increasing temperatures, indicating that O_2_ is essential for SCR reactions.

### 2.4. Transient SCR Activity Test Experiments

**Figure 9:**
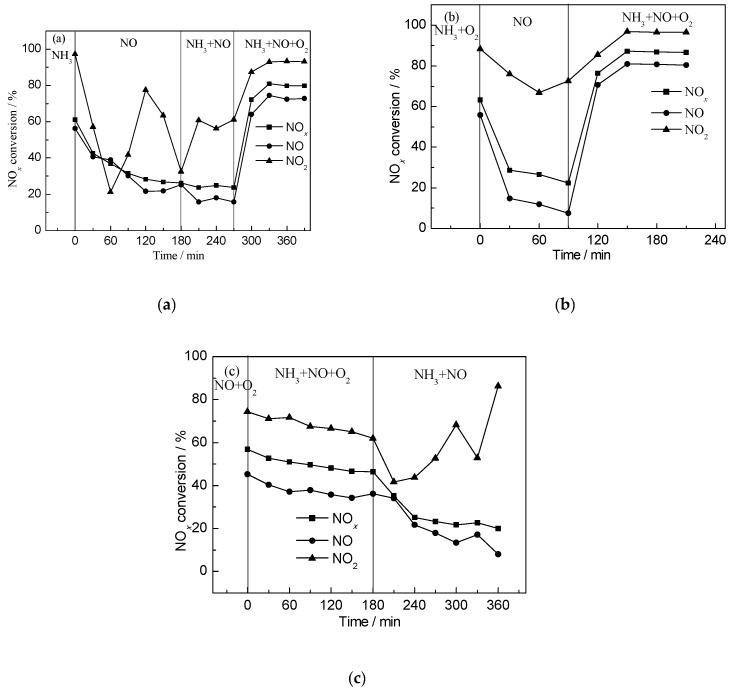
Transient SCR (selective catalytic reduction) activity tests (250 °C) under different pre-adsorption conditions: (**a**) pre-adsorption of NH_3_; (**b**) pre-adsorbed of NH_3_ and O_2_ and (**c**) pre-adsorption of NO + O_2_.

As shown in Figure 9a, NO was introduced after the pre-adsorption of NH_3_ for 2 h. The initial conversion of NO*_x_*, NO, and NO_2_ was 61%, 56%, and 97%, respectively. With a steady flow of NO*_x_*, adsorbed ammonia was consumed gradually and the conversion of NO*_x_* and NO decreased, while NO_2_ conversion went down-up-down. According to the in situ DRIFTS results, it might be that NO_2_ is easier to be adsorbed on the catalysts surface, thus leading to the decrease of NH_3_ adsorption; the conversion of NO_2_ dropped correspondingly. Until NH_3_ was completely consumed, NO_2_ started to be adsorbed on catalysts and the conversion went up, and decreased again after adsorption saturation. By feeding NH_3_ and NO simultaneously, the conversion rate of NO*_x_* and NO was lower than if only NO was fed, suggesting that NO_2_ occupied active reaction sites resulting in its poor performance. In the case of feeding NH_3_ and NO at the same time, we found that all the three conversion rates showed the same trend, namely, that the conversion rate reduced after the first rose, which is associated with the promotion of NH_3_ for SCR reaction. When the three gases: NH_3_; NO; and, O_2_ were fed synchronously, the NO*_x_* conversion rate reached a stable level of 80%. Simultaneously, the conversion rate of NO and NO_2_ stabilized at 73% and 92%, respectively. What is more, both conversion rates obviously increased, indicating that NO_2_ was easier to be reduced. We can conclude that O_2_ was essential for the SCR reaction.

In Figure 9b, NO was introduced after NH_3_ and O_2_ was pre-adsorbed for 2 h. It was obvious that the conversion rate of NO*_x_* reduced compared to Figure 9a. However, the conversion rate of NO_2_ increased and the conversion rate of NO decreased. This might have been caused by the pre-adsorbed O_2_ reacting with NO and producing NO_2_, which was easier to react with, and be adsorbed by, the catalysts. The conversion rate increased rapidly when NH_3_, NO, and O_2_ was present at the same time.

In Figure 9c, after the pre-adsorption of NO + O_2_ for 2 h, the denitration efficiency declined continuously with the existence of NH_3_, NO, and O_2_. When introducing NH_3_ and NO together, the conversion rate of NO*_x_* and NO went down-up-down, while the conversion rate of NO_2_ went down-up-down-up. This could be ascribed to the oxygen storage-release capacity of CeO_2_. NO adsorbed on the catalysts, reacted with O_2_, and produced NO_2_, resulting in the ascended e-conversion rate of NO_2_. When O_2_ was completely consumed, the conversion rate went down again. In this process, the SCR reaction was very weak. As a result, most of the NO_2_ adsorbed on the catalysts, so its conversion rate went up. After this, O_2_ reacted with NO and produced more NO_2_, and its conversion rate declined after the adsorption of NO_2_ was saturated. When O_2_ reacted with NO completely, NO_2_ occupied the adsorption sites of O_2_, leading to the conversion rate going up. The combined effect of the NO_2_ and NO conversion rate resulted in the conversion of NO*_x_* going down-up-down-up.

### 2.5. Low-Temperature SCR Reaction Pathway

The above analyses of in situ DRIFTS have demonstrated the relatively high ability of 1 wt. % V_2_O_5_-0.2CeO_2_/TiO_2_-ZrO_2_ catalyst on NH_3_ adsorption and oxidation. At the reaction temperature (250 °C), the Lewis acid sites were much more stable than were the Brønsted acid sites and the quantity of coordinated NH_3_ was larger than that of the NH^4+^ ions. The gaseous NH_3_ was adsorbed on the catalytic surface, followed by a reaction with the gas phase NO to form the intermediate of NH_2_NO, which was unstable and would decompose into N_2_ and H_2_O (Eley–Rideal mechanism). Based on the combination of in situ DRIFTS experiments and transient SCR activity tests, the mechanism of NH_3_-SCR reaction over V_2_O_5_-CeO_2_/TiO_2_-ZrO_2_ catalysts are mainly as followed:

(1) $$O_{2}{+2}^{*}\to {2O}^{-*}\left(*:\mathrm{surface}\;\mathrm{activesites}\right)$$

(2) $${\mathrm{NH}}_{3}\left(g\right)\overset{{\mathrm{Ce}}^{4+}}{\to }{\mathrm{NH}}_{3}\left(\alpha \right)\left(\mathrm{Lewis}\;\mathrm{acid}\;\mathrm{site}\right)$$

(3) $${\mathrm{NH}}_{3}\left(\alpha \right)+O^{-*}\to {\mathrm{NH}}_{2}\left(\alpha \right)+\mathrm{OH}\left(\alpha \right)$$

(4) $${\mathrm{NH}}_{2}\left(\alpha \right)+\mathrm{NO}\left(g\right)\to {\mathrm{NH}}_{2}\mathrm{NO}\left(\alpha \right)\to N_{2}\left(g\right){+H}_{2}O\left(g\right)$$

## 3. Materials and Methods

### 3.1. Catalyst Preparation

The Ti-Zr support (molar ratio of Ti:Zr = 1:1) was prepared by a co-precipitation method. Typically, an equal molar amount of TiCl_4_ solution and ZrOCl_2_·8H_2_O was dissolved in the deionized water. NH_3_·H_2_O solution was dropped into a stoichiometric solution of TiCl_4_ and ZrOCl_2_·8H_2_O with steady stirring until the pH reached 10. The obtained precipitation solution was aged in air for 24h at room temperature, and then washed with deionized water until the supernatant was free from Cl^−^. Subsequently, the resulting paste was dried at 110 °C for 12 h and then calcined at 450 °C for 4 h in a muffle stove.

1 wt. % V_2_O_5_-0.2CeO_2_/TiO_2_-ZrO_2_ samples were prepared by the step-by-step impregnation of Ti-Zr and CeNO_3_∙6H_2_O (Ce/Ti = 0.2, molar ratio). The obtained mixture was stirred for 2 h at 25 °C, and then for about 4 h at 85 °C until the water boiled away. The resulting precipitate was dried at 110 °C for 12 h, followed by being calcined at 450 °C for 4 h in a muffle stove to obtain intermediate CeO_2_/Ti-Zr, which was then impregnated with a NH_4_VO_3_ solution. The obtained mixture was dried and calcined in the same process of preparing CeO_2_/Ti-Zr samples to finally acquire 1 wt. % V_2_O_5_-0.2CeO_2_/TiO_2_-ZrO_2_ samples.

### 3.2. In situ DRIFTS Experiments

In situ DRIFTS investigations were carried out on a Nicolet 6700 spectrometer (Thermo Electron Corporation, Waltham, MA, USA), running in the wavenumber range of 400–4000 cm^−1^ at a resolution of 4 cm^−1^. A thin, intact and self-supporting wafer of adsorbents were prepared and mounted inside a high temperature cell (HTC-3, Harrick Scientific Corporation, Ithaca, NY, USA). Prior to each experiment, the catalyst was heated to 400 °C under an N_2_ atmosphere for 1h to remove any adsorbed species, then cooled down to the reaction temperature. The background spectrum was recorded in N_2_ flow and was automatically subtracted from the sample spectrum during the experiment. Then the N_2_ flow was switched to a stream containing one or more reactants, such as NH_3_, NO, and O_2_. In situ DRIFTS experiments included transient response and steady-state response experiments. It should be noted that new catalyst samples pretreated under the same conditions and were used in each in situ DRIFTS experiments.

### 3.3. Transient SCR Activity Tests

As shown in Table 1, in order to coordinate the in situ DRIFTS experiments, catalyst activity test experiments were designed under different conditions of feeding gases. A total of 0.3 g of catalyst (screening through 40 to 60 mesh sieve) was tested on a fixed-bed quartz tube reactor (Nanjing University of Technology, Nanjing, China)with an internal diameter of 7 mm at the temperature of 250 °C. The total flow rate was 100 mL/min, which was pre-mixed in a gas mixer to obtain the simulated gas containing 0.08% NO, 0.08% NH_3_, and 5% O_2,_ with a balance of N_2_, NO, NO_2_, and NO*_x_* in the outlet, which was continually monitored by a flue gas analyzer (Testo 330-2 LL, Shanghai, China). Typically, during the experiments, about 5% NO was converted to NO_2_. In other words, 5% NO*_x_* existed in the form of NO_2_.

## 4. Conclusions

In situ DRIFTS experiments and transient SCR activity tests were used coordinately to observe active and intermediate species and to describe the possible reaction path of 1 wt. % V_2_O_5_-0.2CeO_2_/TiO_2_-ZrO_2_ at low temperature. The results are as followed:

(1) NH_3_ held a dominant position in the competitive adsorption between NH_3_ and NO. Transient SCR activity tests showed that the NH_3_ pre-adsorbed catalyst exhibited better SCR activity than its NO*_x_* pre-adsorbed counterpart.
(2) NO might be adsorbed on the catalyst surface and be converted to monodentate nitrite and nitrate species, which is more obvious in the presence of O_2_, and dramatically restrains the adsorption of NH_3_, hindering the SCR reaction.
(3) More acid sites and reaction intermediate species -NH_2_ at lower temperatures mainly led to the higher activity of the V_2_O_5_-0.2CeO_2_/TiO_2_-ZrO_2_ catalyst.
(4) Transient SCR activity tests and steady-state response experiments both confirmed that NH_3_-SCR activity was enhanced by the presence of O_2_. NH_3_ adsorption intensity had no obvious difference, whether NO or O_2_ was introduced or not, indicating that the adsorption and consumption of NH_3_ was in dynamic equilibrium, which promoted SCR reaction.
(5) NH_3_-SCR reaction over 1 wt. % V_2_O_5_-0.2CeO_2_/TiO_2_-ZrO_2_ catalyst mainly follows the E-R mechanism.

## Figures and Tables

**Table 1:** The working conditions of transient SCR activity tests.

Gas Composition	1	2	3	4
I	NH_3_	NO	NH_3_ + NO	NH_3_ + NO + O_2_
II	NH_3_ + O_2_	NO	NH_3_ + NO + O_2_	-
III	NO + O_2_	NH_3_ + NO + O_2_	NH_3_ + NO	-
